# Effect of Interface Structure on Mechanical Properties of Advanced Composite Materials

**DOI:** 10.3390/ijms10125115

**Published:** 2009-11-25

**Authors:** Yong X. Gan

**Affiliations:** Department of Mechanical, Industrial and Manufacturing Engineering, College of Engineering, University of Toledo, 2801 W Bancroft Street, Toledo, OH 43606, USA; E-Mail: yong.gan@utoledo.edu; Tel.: +1-419-530-6007; Fax: +1-419-530-8206

**Keywords:** composite materials, adhesive bonding, interface, nanostructure, self-healing, mechanical property, nonlinear damage model

## Abstract

This paper deals with the effect of interface structures on the mechanical properties of fiber reinforced composite materials. First, the background of research, development and applications on hybrid composite materials is introduced. Second, metal/polymer composite bonded structures are discussed. Then, the rationale is given for nanostructuring the interface in composite materials and structures by introducing nanoscale features such as nanopores and nanofibers. The effects of modifying matrices and nano-architecturing interfaces on the mechanical properties of nanocomposite materials are examined. A nonlinear damage model for characterizing the deformation behavior of polymeric nanocomposites is presented and the application of this model to carbon nanotube-reinforced and reactive graphite nanotube-reinforced epoxy composite materials is shown.

## 1. Introduction

Advanced composite materials have the unique combination of outstanding mechanical properties of matrices and reinforcements. The reinforcement/matrix interface in composite materials forms in manufacturing processes and determines the performances of the composite materials. Some reinforcements may not be compatible with matrices in view of their physical and/or chemical properties, which causes premature failure of the composites. For example, ultrahigh molecular weight polyethylene (UHMWPE) fibers have poor wettability with epoxies. As a result, the interface bonding strength between the fibers and polymer matrices is very low. Recently, development of nanofiber modified matrices containing reactive graphitic nanofibers (r-GNFs) has been proposed to promote the wetting of the matrices to certain types of fiber reinforcements. In this paper, the effect of interface structures on the mechanical properties of fiber reinforced composite materials is discussed. The wettability of UHMWPE fibers with different epoxy matrices including a surface modified carbon nanotube-containing epoxy and a pure epoxy is presented. How to change the interface structures to reduce the contact angle between the epoxies and the fibers is described. Finally, the nonlinear damage model for evaluating the mechanical property change associated with the interface damage is presented.

## 2. Hybrid Composite Materials

Composite materials are designed to have a combination of the properties of each of the components. There are several types of composite materials, including particle-reinforced, fiber-reinforced composite materials, *etc.* Hybrid composite materials containing continuous fiber reinforced plies and metal layers are special composite materials because of their high specific strength, high specific modulus, excellent electromagnetic shielding characteristics and very good high-cycle fatigue property. Typical examples of such composites include carbon fiber reinforced aluminum foams or laminates (CARE) [1], Kevlar fiber reinforced aluminum hybrid composites (ARALLs) [2]. These composite materials consist of alternating layers of metal sheets and fiber reinforced epoxy composites. The unique properties of the fiber reinforced epoxy composites are retained and the materials are immune to environmental attack due to the incorporation of the sandwiched metal layers. The metal layers are also responsible for providing high shear strength. Therefore, applications of such hybrid composite materials in aerospace, electronics and automotive industries have been considered [3].

Hybrid composite materials/structures are frequently subjected to thermal and mechanical fatigue loading. Aside from external mechanical loadings, thermal effect is identified as an important factor that determines the stress distribution in composite materials. During the curing process, adhesively bonded composite/metal laminate structures are held at elevated temperatures over 120 °C, very high residual stresses could build up because of the difference in coefficients of thermal expansion (CTE) for different materials. The CTE of aluminum is about 2.36 × 10^−5^/°C and for polymers it is higher than 1.05 ×10^−4^/°C [4]. This thermal mismatch results in delamination or debonding of hybrid composite materials, which facilitates fatigue crack growth in the polymer/metal interface. Thermal cyclic stresses can also be generated from the fluctuation of ambient temperatures. For example, the change in environmental temperatures is obvious when an aircraft travels across different continental regions or varies altitudes. For electronic devices, the temperature variation associated with the power on/off can reach as high as several tens of degrees. Therefore, the stress state in a hybrid composite material is not only dependent on service conditions, but also affected by the materials processing parameters. The overall stress distribution influences the fatigue crack growth behavior and the durability becomes an increasing concern. In many cases, fatigue damages in the interface region account for the majority of failures of materials.

Extensive research on the mechanical properties of metal/polymer structures has been performed [5–18]. Sekercioglu, Gulsoz and Rende [5] found that the strength of adhesively bonded cylindrical components is affected by various factors including diametrical clearance, assembly type, material properties, operating temperature, loading type, and surface roughness, among which the thickness of the interface is the most significant factor. As the bonding clearance increases, significant decreases were observed in the static and dynamic strengths. Based on the studies of carbon fiber reinforced composites bonded to steel plates, Oehlers, Liu and Seracino [6] revealed that debonding generated by shear deformation is the primary form of failure in adhesively bonded structures. Tantikom, Aizawa and Mukai [7] reported their work on symmetric to asymmetric deformation transition in regular cellular materials. The effect of adhesive bonding on such deformation mode transition was investigated under quasi-static in-plane compression loading conditions. Based on an elastic-plastic formulation through finite element (FE) analysis, a computational model was proposed for understanding the effect of various parameters on the deformation mode transition. It was found that the symmetric deformation changes to asymmetric deformation when the nominal compressive strain is increased. Cognard *et al.* [8] studied the behavior of thin adhesive films and tested simple composite assemblies. However, difficulty in modelling the failure of very simple joints was found due to the lack of reliable constituent input data.

Improvement of the reliability of hybrid composite materials relies on the enhancement of polymer/metal interface bonding. Various surface treatments including alkaline etching and acid pickling (applied separately or in combination with phosphoric acid anodizing) [19], plasma processing [20–23], ion beam irradiation [24–26], and coupling agent treatment [27] have been explored to examine the effect of pre-treatment on the adhesive bonding between metals and polymers. It is found that the presence of oxide and small molecules such as water in the interface region is responsible for the degradation of bonded joints [19]. Recent studies have shown that the chemical bonding at metal-polymer interface plays an important role in adhesion. Thus, the interfacial bonding and subsequent adhesion are directly influenced by the way that the interface is formed. David, Lazar and Armeanu [28] studied an aluminum/polymer joint where a thin and uniform metal sodium layer was coated on the polymer surface. The nature of the bond formation at the metal/polymer interface was investigated in view of compound formation and charge transfer between sodium and the polymer. A bonded joint was tested in terms of its strength, thermal resistance and tightness to show the interfacial properties.

Underhill and Rider [29] investigated hydrated oxide film formation on aluminum alloys immersed in warm water. Porous oxide structure was found due to the growth of hydrated oxide films on 2024 and 7075 aluminum alloys immersed in deionized water, at the temperatures of 40~50 °C for periods up to a couple of hours. In contrast with film growth studies reported for pure aluminum, the alloy systems do not appear to show an incubation period prior to hydrated oxide growth. Various characterization techniques were applied to study the properties of the oxide structure including Fourier Transform Infrared Spectroscopy (FTIR), weight gain measurements, high resolution Scanning Electron Microscopy (SEM) and Atomic Force Microscopy (AFM). It was found that the films formed at 50 °C are much thicker than those formed at 40 °C. However, the porosity of the films appears to be comparable at both temperatures. The research has suggested that a porous oxide structure is likely to be very suitable for adhesive bonding because of the increase in interface area of nanoporous structure, which results in the high shear loading capability. However, the interface nanostructure remains to be revealed by further systematic study.

## 3. Polymer/Metal Bonded Composite Structures

In addition to making high performance hybrid composite materials, bonding composites to metals is an effective method for repairing structural defects, maintaining the load carrying capability, and extending the service life of metallic structures [30–50]. Adhesive bonding offers various advantages [43,46] and the repair of defective structures with composites has found various applications. One of the challenging aspects related to metal/polymer bonding is the long-term durability of the interface between the composites and the substrate structure. To ensure a reliable and durable bonding, materials design, stress analysis of the structures, and optimization of processing conditions have been extensively studied.

In view of materials selection, boron-epoxy, carbon-epoxy and graphite-epoxy composites have been used to form polymer composite/metal bonded structures. The thermal expansion coefficient of boron-epoxy closely matches that of most metals, which makes it one of the best candidate composite materials for structural bonding applications. Experimental work [51] showed that restoration of more than 90% strength of the original laminate is possible with the use of bonded composites. For a typical bonded configuration, a large peak in the adhesive shear strain occurs at the end of the patch. Currently, uniform stepping of the multi-layer laminate is used for bonded structures because the reduced peak adhesive shear strains lead to a smoother transition of load from metals to composites. The increase of consolidation pressure enhances the bonding of the interface.

The performance of the adhesive plays a key role in the determination of the bonding strength. Many adhesives were used to form composite/metal-bonded structures [52–56]. The adhesive bonding process requires curing at elevated temperatures, which leads to thermal residual stresses due to the thermal expansion mismatch between composite materials and metals. To decrease the thermal residual stresses in the structure, the curing cycles of high temperature adhesives were modified to temperatures lower than their standard curing conditions [57]. The optimized curing conditions were determined for each adhesive based on the bonding strength. One of the critical steps in the bonding procedure is the substrate surface preparation. To obtain sufficient shear strength for adhesive bonding, the bonding area should be thoroughly cleaned and abraded before the preparation of bonding patches. The procedures for aluminum alloy substrate preparation were given in [58,59].

Analytical work on adhesive bonding can be found in many literatures, for example [60–65]. Rose’s analytical solution [61] was used by Muller *et al.* [66]. It is shown that the stress intensity factor *K* estimated using the Rose model provides a reasonably good connection with observed d*a*/d*N*, where *a* is the crack length and *N* is the cycle of fatigue loading. Many numerical analysis techniques, such as finite element method (FEM) [67–81], boundary element method (BEM) [82–84], and finite element alternating method (FEAM) [85,86], have been proposed to the stress analysis of repaired structures. Atluri, Chow and Wang [87] applied the FEAM to model composites bonded to metals. The numerical results were compared with the experimental data.

The improved durability of bonded composite/metal structures was validated under practical application conditions. Fatigue property of notched composite/metal specimens subject to cyclic loadings was tested [88–90]. The fatigue life of cracked aluminum bonded with carbon or boron-fiber composites was extended 60 to 100 times. The fatigue life increased three times with composite patches over that of riveting metal patches to the same honeycomb panels [91]. The repair is capable of restoring residual static strength and reducing the crack growth rate by approximately two orders of magnitude [92]. Both static strength and fatigue life of the bonded plates have been significantly increased for the bonded composite patches of aircraft structures [93]. The size of adhesive-bonded region can be estimated empirically [94]. It is found that fatigue crack growth retardation is better achieved by bonding full patches to both faces of a specimen and by using a thicker composite laminate [95]. Hastie *et al.* [96] conducted photoelastic experiments on a center-cracked tension panel. The bonded repair arrested the crack for a significant number of load cycles (N = 10^4^~10^5^) and reduced the crack growth rate significantly. Jones *et al.* [97] investigated the ability of a bonded-composite doubler to restore the fatigue performance of lap joints containing multi-site damages. In all cases the repaired specimens survived more than 200,000 cycles without failure. Tay *et al.* [98] showed that if pure metallic specimens failed at about 10,000 cycles, the boron-epoxy bonded specimens survived more than 200,000 cycles. Environmental effects on the fatigue of a toughened epoxy adhesive were studied [99]. The results indicate that high temperature and high humidity tend to facilitate interface debonding and accelerate the fatigue crack growth.

The results from previous research provide useful information about materials processing and mechanical property characterization. Although metal/polymer hybrid composite materials and metal/composite repairs have been used in various fields, the relatively weak bonding between metal/polymer interfaces still remains a problem to be solved. Studies on the interface architecturing have recently been considered. Previous work fails to consider the bonding nature and the mechanical response of the metal/polymer interface at multiscale length levels. The design and processing methodologies are limited to using the conventional techniques for fabricating fiber reinforced composite materials. Since fiber reinforced polymer/metal hybrid composite materials or repair structures contain special polymer/metal interfaces, which are highly heterogeneous in chemical, physical and mechanical properties, novel interface design methodologies are introduced to improve their performances.

## 4. Nanoarchitectured Interfaces in Polymeric Composites

**Figure 1.**
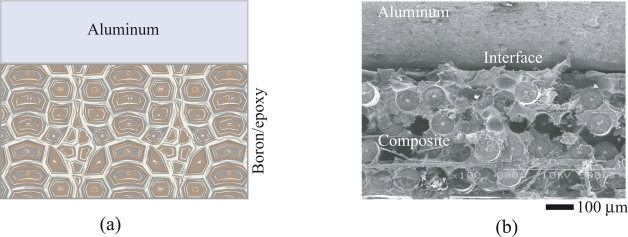
Composite/metal interface as the site for failure: (a) schematic of a boron/epoxy bonded to aluminum substrate to form a hybrid composite structure; (b) SEM image showing the interface debonding feature.

The weakest link in hybrid composite materials or adhesive bonding structures lies in the metal/polymer interface region. As shown in Figure 1, the failure of the boron fiber/epoxy/Al hybrid composite occurs mainly by interface delamination. Figure 1(a) is the schematic of the boron fiber/epoxy bonded onto aluminum layer to form a hybrid composite structure. Figure 1(b) is a scanning electron microscopic (SEM) image showing the fracture surface morphology of the hybrid composite. From this SEM image, the “composite/Al” interface is identified as the initiation site for the failure of the hybrid composite/structure. Conventionally available adhesives are susceptible to shear loadings because of their brittleness and weak physical bonding to metals, leading to low interface shear strength. Therefore, in order to improve the performances of metal/polymer hybrid composite materials, or bonded structural repairs, the interface bonding must be enhanced. New interface design strategies are considered to meet with the requirements. For example, nano-architecturing the interface between metal and polymer is one option. A high performance interface prototype is considered for the aluminum/epoxy system and the same principle may be extended to other metal/polymer systems.

### 4.1. Formation of nanoporous surface structures

**Figure 2.**
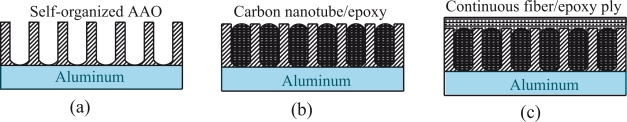
Schematic diagrams for nanoarchitecturing fiber reinforced epoxy/aluminum interface: (a) fabrication of AAO; (b) impregnation of active multi-wall carbon nanotubes/epoxy and adding nanoscaled spheres containing crack-healing resin and cure agent; (c) laminating continuous fiber reinforce epoxy plies and vacuum curing.

Self-assembled nanoporous feature in the form of anodic aluminum oxide (AAO) was prepared first on the surface of aluminum. Anodic aluminum oxide (AAO) nanoscale pores have recently been studied due to their peculiar self-organizing capability [100,101]. A two-step anodic oxidization method was used to obtain nanopores with uniform size and thin barrier layer. Aluminum alloy thin sheets were anodized on both sides using a regulated DC power supply. Before anodizing, the aluminum plates were degreased first in trichloroethylene for 2 h, followed by ultrasonic cleaning for 10 minutes in acetone. Then the samples were rinsed with methanol and distilled water, respectively. After that, the aluminum plates were etched in a 5.0 M NaOH solution at 60 °C for 5 minutes and subsequently rinsed with distilled water. Anodizing was performed in 0.3 M H_2_C_2_O_4_ at ambient temperature. This first anodizing will take about 1 h. After the first anodizing, a strip-off process was carried out in a mixture solution of H_3_PO_4_ and H_2_CrO_4_. The exposed and well-ordered concave patterns on the aluminum substrate act as a self-assembled mask for the second anodizing process. The second anodizing took about 2 h. After the second anodizing, AAO templates with uniform nanopores were obtained. The depth of the nanopores was controlled in the range of 500–600 nm, and the diameter of the pores was in the range of 80–100 nm. The AAO on Al plate is schematically shown in Figure 2(a).

The nanostructured interface and hybrid composite materials are shown in Figure 2(b),(c). It is noted that the thickness of the AAO can be controlled by the oxidization time. The initially formed nanopores have the diameter about 20 nm. Pore expansion can be precisely controlled by the anodic oxidization parameters and the chemical treatment in a warm H_3_PO_4_ solution. The temperature, treatment time, and the concentration of the H_3_PO_4_ solution determine the final size of the nanopores, which may be varied between 20 nm and 200 nm. In order to control the volume fraction of the AAO nanopores, photolithography procedure may be introduced using a photo mask with microscale patterns. After the photolithography step, the surface of aluminum plate was covered by micropatterns, and oxidization to form AAO only occurs in the region without photoresist. Therefore, selective growth of AAO is achieved and the volume fraction of the AAO can be precisely controlled. The nanoscale pore formation on the surface of other metallic alloys including stainless steel and titanium alloys is also possible. These materials can be electropolished in a solution containing both HF and HNO_3_ before manufacturing nanopores. By using different electrolytes and changing the electrical parameters in the anodic oxidization processes, passive films consisting NiO and Cr_2_O_3_ can be generated on the surface of stainless steels, while TiO_2_ barrier film can be obtained on the surface of Ti-based alloys.

### 4.2. Addition of active carbon nanotubes

Active carbon nanotubes with functional groups [102] were added into an epoxy matrix, and the modified epoxy resin containing active carbon nanotubes was introduced into the nanopores of the AAO. The active functional groups help to form strong chemical bonding both between carbon nanotubes (CNTs) and epoxy, and between epoxy and AAO. Moreover, the interface bonding is enhanced by the large specific area of the AAO, resulting in a significant improvement on the interface strength that is typically unattainable in conventional adhesive/bonding techniques.

**Figure 3.**
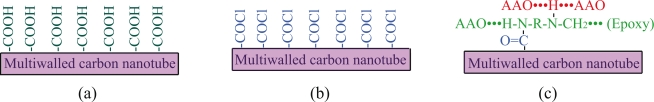
Illustration of preparation of active carbon nanotubes: (a) nitric acid refluxing, (b) converting –*COOH* groups into acid chloride functional groups, (c) bonding enhancement between carbon nanotubes and AAO; carbon nanotubes and amine groups in epoxy backbones.

Multi-walled and single-walled carbon nanotubes are used as additives in polymer materials to enhance the mechanical performance of the polymeric composite materials [103–116] because carbon nanotubes possess special properties [117]. Carbon nanotubes can be produced in relatively large quantities at reasonable costs using metal catalysts and either ethylene or carbon monoxide as the carbon source [118]. The structure of carbon nanotubes can be controlled through the catalyst and thermal conditions used in production. By appropriate surface treatment, carbon nanotubes present a unique, active surface so that the carbon nanotube/polymer covalent bonding can be established [119,120]. The commercially available multi-walled carbon nanotubes (MWCNTs) produced by Ahwanhnee Technology, Inc. for nano-architecturing the interface of hybrid composites were used. These tubes contain about 10 to 70 graphene layers. The diameter of the MWCNTs is in the range of 10~50 nm and the length of the tubes is in the range of 1–10 microns. Surface treatment was performed in nitric acid so that the surface of the tubes are rich in functional group of –*COOH* as shown in Figure 3(a). The next step includes the reaction with thionyl chloride to convert the surface –*COOH* group to acid chloride functional groups as shown in Figure 3(b). The carbon nanotubes containing acid chloride functionalities are very active to the amine cure agent for epoxy. The active carbon nanotubes were mixed with epoxy and the curing agent, as shown in Figure 3(c), secondary bonding type in the form of hydrogen bond between the AAO and the cross-linked epoxy and amine can be established. Therefore, the active carbon nanotubes are helpful to improve the interface bonding between the carbon nanotube/epoxy and epoxy/AAO. As a result, the metal/polymer interface bonding is improved.

### 4.3. Addition of nanoscale self-healing capsules

Structural composites are susceptible to damage in the form of microcracks, which are usually initiated in the reinforcement/matrix interface area or deeply within the structure where detection is difficult and repair is almost impossible. Regardless of the application, microcracks are the precursors to structural failure and the ability to heal them will enable structures with extended lifetime and less maintenance. The biologically inspired, self-healing composites possess great potential for solving some of the most challenging problems of composite structural materials. Many previously reported self-repair techniques require some manual intervention [121]. Automatically healing of cracks may be accomplished by incorporating a microencapsulated healing agent and a catalytic chemical trigger in the matrix [122]: An approaching crack ruptures embedded micro- or nanocapsules, releasing a healing agent into the crack plane through capillary action. Solidification of the healing agent is then triggered by contacting with an embedded catalyst. Therefore, bonding and healing the crack faces happen.

**Figure 4.**
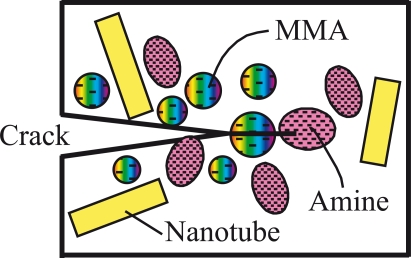
Illustration of active healing a crack by releasing self-curing polymers.

Nanosize capsules containing self-curing resin may be added into the epoxy matrix so that active healing of the damage/small cracks in the composite materials can be achieved. Under interface debonding and crack propagation conditions, the nanoscale spheres in front of the crack tip will be broken and the curable resin such as methacrylate (MMA), and the curing agent, amine, are released as shown in Figure 4. Self-repairing effect may be achieved upon curing of the agent and the crack surfaces are bonded. Thus under the above described design, the interface structure becomes an active nanosystem, which has the function of self-healing once damage occurs. The advantage of designing such a functional active nanosystem is that the interface bonding can be substantially enhanced. Continuous fiber plies can then be stacked on the top of the multi-layered structure. Upon curing under vacuum conditions, a fiber reinforced epoxy/aluminum hybrid structure with nano-architectured interfaces is obtained. Due to the enhanced interface bonding, the novel hybrid composite structure is expected to receive wide applications, such as load carrying components with both high tensile strength, shear strength, stiffness and excellent fatigue property. The novel interface nano-architecturing strategy is also suitable for repairing defected metallic structures and can be extended to other metal/polymer systems.

Self-healing polymer research has become a very active field. For example, the rheological and kinetic behaviors of self-healing agents have been studied to evaluate how fast the healing agents polymerize [123]. Urban [124] outlined the stimuli-responsive polymer networks that possess multiple functions in signaling, reorganizing and self-healing. Kavitha and Singha [125] prepared thermally amendable and self-healing homo- and copolymers by atom-transfer polymerization. Crack-healing in carbon-based materials at high temperatures has been studied [126]. Iyer and Lyon [127] reported their work on self-healing of colloidal crystals. In fiber reinforced polymeric composite materials, matrix failure is a problem. Recently, the healing behavior of a matrix crack on a carbon fiber/mendomer composite has been studied [128]. It is possible to have self-healing functions in different forms of materials or structures, for example, in films [129], coatings [130] and high elasticity block copolymers [131]. Pegoretti [132] reviewed the methods for preparing self-healing polymers and composites. Self-healing shows the increasing importance and has found various applications, especially in military [133]. Other applications such as for recycling of polymers [134] have also been studied.

## 5. Nonlinear Damage Model for Polymeric Nanocomposites

Since polymeric composites display progressive damage under external loads, nonlinearity is associated with the deformation of such materials. Nanofiber reinforced epoxies can change the wettability between the matrix and several types of reinforcements such as high strength polyethylene fibers and carbon fibers [135]. It is also possible to tailor the values of thermal expansion coefficient by controlling the content of the nanofiber in the epoxies. Therefore, nanoreinforced polymers could alleviate the residual stress problems in composite materials. For nanoreinforced composites, because of the increased toughness, nonelastic behaviors will dominate the deformation, fracture and failure processes [136,137]. Thus the prevailing linear damage models have to be modified. Nonlinear damage should be considered in nanoreinforced polymeric composites.

The nonlinear behavior of epoxies and their nanocomposites is considered as the combination of various damage events such as interfacial debonding, fiber breakage, micro/nanoscale crack propagation within the materials, and micro-yielding of the matrices. It is assumed that some of these damage events occur at low stress levels and the accumulation of damage can be revealed by the non-linear regions on stress~strain curves of the nanocomposites.

### 5.1. Modeling the nonlinearity

Pervin, Zhou, Rangari *et al.* [138] proposed a nonlinear damage model to to describe the stress~strain relationship of nanocomposites. The nanocomposite materials containing the SC-15 epoxy resin and carbon nanofibers (CNFs). The contents of the nanofiber are 1 wt%, 2 wt% and 4 wt%. The characteristic parameters in the model include Young’s modulus of the materials, Weibull scale parameter and Weibull shape parameter. The nonlinear relationship between the stress and strain for the epoxy nanocomposites is expressed as

(1) σ=εE(1−ξ)

where σ is the stress, *E* is the Young’s modulus,ε is the strain, and ξ is a damage factor. ξ is considered as a Weibull function

(2) $$\xi =P(\varepsilon ,E)=1-\text{exp}\left[-{\left(\frac{\varepsilon E}{\sigma_{\circ }}\right)}^{m}\right]$$

where *P* is the cumulative probability of failure, σ_o_ is the Weibull scale parameter, and *m* is the Weibull shape parameter. The Weibull scale parameter σ_o_ is the measure of the nominal strength of the composite material. With the increase in the value of σ_o_ the average strength of the composite increases. The shape parameter *m* is the measure of the scattering in strength data which reveals the dispersion of the distribution in micro/nanoscale crack length. The higher the value of *m* is, the less scattering of the composite strength; in other words, a larger *m* means more uniform and narrow flaw distribution.

Substituting Equation (2) into Equation (1) yields

(3) $$\sigma =\varepsilon E\text{exp}\left[-{\left(\frac{\varepsilon E}{\sigma_{\circ }}\right)}^{m}\right]$$

Rearranging Equation (3) gives

(4) $$\frac{\sigma }{\varepsilon E}=\text{exp}\left[-{\left(\frac{\varepsilon E}{\sigma_{\circ }}\right)}^{m}\right]$$

Taking double logarithms on both sides of Equation (4) produces

(5) $$\text{ln}\left[\text{ln}\left(\frac{\sigma }{\varepsilon E}\right)\right]=m\text{ln}(\varepsilon E)-m\text{ln}(\sigma_{\circ })$$

If the nonlinear behavior of the nanocomposites satisfies the Weibull distribution, Equation (5) represents a straight line. From the slope and the intercept of the line, the values for the two parameters, *m* and σ_o_ can be determined.

### 5.2. Application of the nonlinear damage model

Jana, Zhong and Gan [139] applied the above nonlinear model to characterize the flexural deformation behavior of reactive graphite nanofibers (r-GNFs) reinforced epoxy nanocomposite materials. It is reported that the predicted stress *vs.* strain relations for the nanocomposites with different r-GNFs contents by the non-linear damage model are in agreement with the testing results. The *m* and σ_o_ values for different composites calculated from experimental data using the nonlinear model of Equation (5) are listed in Table 1. From three point bending tests, it is found that the r-GNF/E-2 nanocomposite containing the epoxy and 0.3 wt% reactive graphite nanofibers (r-GNFs) has the average flexural strength around 166 MPa, while the pure epoxy specimens have an average flexural strength only around 132 MPa. For calculating the flexural strength, the following expression, Equation (6), from the beam theory was used

(6) $$\sigma_{u}=\frac{3FL}{2wt^{2}}$$

where *F* is the peak load in the three point bending tests, *L* is the span of the specimens, *w* is the width of the specimens and *t* is the thickness of the beam specimens. The strain at failure was given by Equation (7)

(7) $$\varepsilon_{u}=\frac{6Dt}{L^{2}}$$

where *D* is the maximum deflection in the three point bending tests.

Referring to the data in Table 1 for the matrix and the r-GNF reinforced nanocomposites, the *m* value for the pure epoxy obtained from theoretical analysis using the nonlinear damage model is the highest, thus the scattering in strength for the epoxy is the lowest. The theoretical flexural stress~strain curve of the pure-epoxy is more linear compared with the experimental result, which implies that the mechanical deformation of the pure-epoxy should not be nonlinear. This is true because the epoxy is less ductile or more brittle than all the other three materials containing the reactive graphitic nanofibers. In contrast, the r-GNF/E-2 material exhibited apparent nonlinearity in the flexural test. The behavior fits well to the non-linear damage model. This can also be confirmed by the fracture surface morphology analysis, which reveals that the r-GNF/E-2 nanocomposite is more ductile than any other material listed in Table 1. The nanofiber reinforced epoxies with enhanced ductility may be used as the matrix for manufacturing high performance hybrid composites or structures. It may also be used as adhesive to make bonded joints with nanostructured interface. As discussed in Section 3., bonding composites to metallic structures is a highly cost-effective method for extending the service life of the structures. Residual stresses due to the mismatch in the coefficients of thermal expansion between metals and composites often cause premature failure of bonded structures [140]. The nanofiber reinforced epoxies as new adhesives may help to alleviate the residual stress problem because they are more ductile than the conventionally used pure epoxy adhesives.

## 6. Conclusions

Reinforcement/matrix interface plays the key role in determining the performance of advanced composite materials. To enhance the interfacial bonding, nanostructures are introduced into composite materials. Formation of nanopores on metal surface can increase the bonding strength of the metal/polymer interface. Surface treated carbon nanotubes are used in preparing nanoreinforced matrices. The nanofiber reinforced epoxies containing reactive graphitized carbon nanotubes as new adhesives can help to alleviate the residual stress problem because they are more ductile than the conventionally used pure epoxy adhesives. Finally, the progressive damage of interfaces in composites can be evaluated by nonlinear models due to the complexity of the deformation and failure processes.

## Figures and Tables

**Table 1.** Properties of nanofiber reinforced epoxy composites (data source: [139]).

Nanocomposite material type	Epoxy	r-GNF/E-1	r-GNF/E-2	r-GNF/E-3
The r-GNF content (wt%)	0	0.20	0.30	0.50
Young’s modulus, *E* (MPa)	2770	3004	3337	3269
Flexural strength, σ*_u_* (MPa)	132	152	166	160
Weibull shape parameter, *m*	26.4	8.3	3.4	4.1
Weibull scale parameter, σ_o_(MPa)	150	211	299	278
